# Transient Leukemoid Reaction from T-Cell Large Granular Lymphocytes Post Autologous Stem Cell Transplant in a Patient Affected by Hodgkin Lymphoma

**DOI:** 10.3390/hematolrep15040058

**Published:** 2023-10-11

**Authors:** Andrea Duminuco, Marina Parisi, Giulio Antonio Milone, Alessandra Cupri, Salvatore Leotta, Giuseppe A. Palumbo, Nunziatina Laura Parrinello, Grazia Scuderi, Anna Triolo, Giuseppe Milone

**Affiliations:** 1 Hematology Unit and Bone Marrow Transplant, A.O.U. Policlinico “G. Rodolico—San Marco”, 95123 Catania, Italy; marinaparisi@hotmail.it (M.P.); alessandracupri@hotmail.it (A.C.); leotta3@yahoo.it (S.L.); lauraparrinello@tiscali.it (N.L.P.); graziascuderi@hotmail.it (G.S.); cit.triolo@libero.it (A.T.);; 2 Division of Hematology with BMT, Istituto Oncologico del Mediterraneo, 95029 Viagrande, Italy; giulio.milone89@gmail.com; 3 Department of Medical, Surgical Sciences and Advanced Technologies, “G.F. Ingrassia”, University of Catania, 95123 Catania, Italy; palumbo.gam@gmail.com

**Keywords:** Hodgkin lymphoma, T-cell large granular lymphocytic leukemia, cytotoxic T-lymphocytes, ASCT, lymphoproliferative disorder

## Abstract

Monoclonal T-cell lymphocytosis has been reported in patients with concomitant autoimmune diseases, viral infections, or immunodeficiencies. Referred to as T-cell large granular lymphocytic leukemia (T-LGLL), most cases cannot identify the triggering cause. Only small case series have been reported in the literature, and no treatment consensus exists. T-cell lymphocytosis may also appear after the transplant of hematopoietic stem cells or solid organs. Rare cases have been reported in patients undergoing autologous stem cell transplant (ASCT) for hematological diseases (including multiple myeloma or non-Hodgkin’s lymphoma). Here, we describe the singular case of a patient who underwent ASCT for Hodgkin’s lymphoma and displayed the onset of T-LGLL with an uncommonly high number of lymphocytes in peripheral blood and their subsequent spontaneous remission.

## 1. Introduction

The occurrence of a monoclonal peripheral lymphocytosis sustained by cytotoxic T-lymphocytes (CTL) is usually known as T-cell large granular lymphocytic leukemia (T-LGLL). According to the WHO classification, it is defined as a heterogeneous disorder characterized by a persistent increase in the number of these large granular lymphocytes in the peripheral blood for at least 6 months, without a clearly identified cause [1]. T-LGLL is a cellular dyscrasia more frequent in elderly patients and often associated with rheumatological conditions (including rheumatoid arthritis), non-hematological tumors, and immunodeficiencies (especially hypogammaglobulinemia) [2,3]. These lymphocytes (normally between 2 and 10 × 10^9^/L), analyzed in the peripheral blood smear, appear large (15–18 μm), with abundant cytoplasm. Azurophilic granules may be present in a reniform or rounded nucleus. Immunophenotypic examination shows a cell population in most cases CD3+, CD7+, CD4−, CD5−, CD8+, CD30−, CD16+, CD27−, CD28−, CD56−, and CD57+ [4]. Clonality is confirmed using the TCR-γ-polymerase chain reaction (PCR), with, in more than 90% of cases, monoclonality for TCR-αβ [5]. A dysregulated apoptotic function is a possible mechanism involved in the pathogenesis of T-LGLL. The activation of some transcription factors and signal transduction pathways (JAK2/signal transducer and activator of transcription 3/Mcl-1, RAS/MAPK, SFK/PI3K/AKT, and sphingolipid signaling pathways) would seem to influence the progression and maintenance of this lymphocytosis [6]. Specifically, the STAT3 gene plays a leading role. The mutation of this gene has been seen to be a driver mechanism in perpetuating the autonomy proliferation of these cells [7]. In any case, the etiology of T-LGLL is unclear, although the most likely hypothesis is that of a dysregulated monoclonal response to a chronic antigenic stimulus [6]. Clinically, the manifestations could be recurrent infections attributable to concomitant neutropenia. From the analysis of a large cohort of 229 patients affected by T-LGLL associated with various pathological conditions in a French study, it was seen that there might be hypogammaglobulinemia (38%) or high serum β2-microglobulin (66%), with bone marrow infiltration in more than 70% of the analyzed samples, but without correlation between the degree of pancytopenia and the level of bone marrow involvement [8].

Considering that T-LGLL is a chronic disease, there are discordant data on the incidence of related deaths, mainly due to infective complications (9 deaths out of 25 cases in a follow-up of just over 2 years vs. a superior median survival of 10 years in 68 patients in two different studies, respectively) [2,9]. The challenge is identifying cases that can benefit from therapy (usually based on steroids and immunosuppressive drugs), instead of a “watch and wait” approach, with erythropoietin or antibiotics and granulocyte colony-stimulating factor in case of febrile episodes. The accepted indications for treatment are moderate–severe neutropenia (neutrophils < 500/mmc and between 500 and 1000/mmc), symptomatic or transfusion-dependent anemia, with concomitant autoimmune conditions that require appropriate therapy [6]. In any case, when considering T-LGLL as an indolent chronic disease, the overall survival at 10 years is ∼70%, with disease-related death due to severe infection [10].

Returning to the possible association with concurrent disorders, autoimmune diseases account for 15–40% of the total. Rheumatoid arthritis, Sjogren’s syndrome, and polymyositis are the most frequent. Other conditions are autoimmune cytopenia, viral infections (HIV, CMV, HCV, and EBV), and neoplasms of both solid and hematological organs (myelodysplasia, B-cell malignancies, and chronic myeloid leukemia) [6,11]. Finally, the possibility of onset in the period after the transplant of solid organ or hematopoietic stem cells (HSCT) was rarely reported [12].

In this case, with an extended follow-up, we report the clinical presentation and outcome of a patient affected by T-LGLL that arose after autologous HSCT for Hodgkin lymphoma.

## 2. Case Presentation

**Figure 1:**
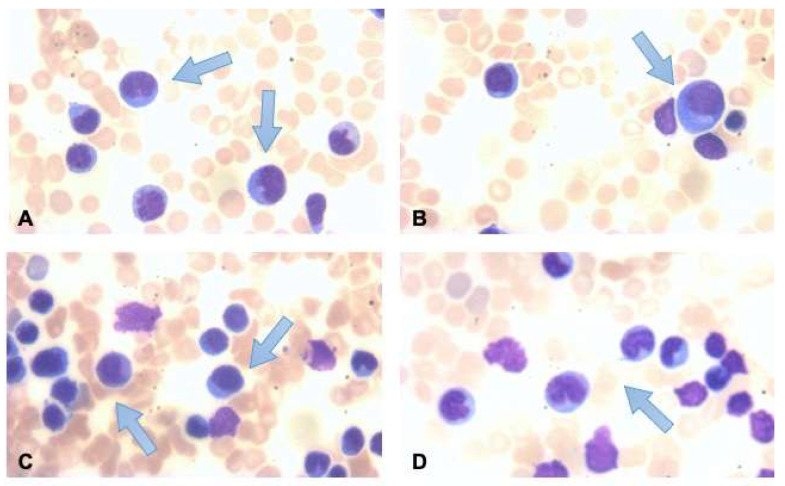
(**A**–**D**) Monomorphic appearance in peripheral blood smear with lymphocytosis, indicated by arrows. Cytotoxic lineage was confirmed via the immunophenotypic study (CD3+, CD8+, TCRαβ+, CD2+, CD5+, CD1a−, CD7±, CD38+, HLA-DR+).

A 47-year-old patient presented to us in January 2011 with supra- and subdiaphragmatic lymph node enlargement. A lymph node biopsy was performed, which found large mononuclear cells CD30+, CD3−, CD79a−, CD3−, CD20− and CD15+, diagnosing him with Hodgkin’s lymphoma (HL), stage Ann Arbor IVA due to the bone marrow involvement. He underwent chemotherapy treatment for this diagnosis according to the ABVD scheme for 8 cycles, followed by radiotherapy on the involved abdominal lymph nodes. A complete response to treatment was achieved, confirmed through CT and PET exams (Deauville 1). Two years after the end of treatment, in September 2013, the patient experienced a recurrence of lymphoma disease, with new localization on both sides of the diaphragm accompanied by mild splenomegaly (about 13 cm). A new inguinal biopsy confirmed relapse of HL, and was also evaluated for a second opinion in another reference center. According to the ESHAP scheme, second-line therapy was administered for 6 cycles. Due to a persistent mild glucose uptake at PET re-evaluation in the spleen, he underwent splenectomy. The subsequent histological examination did not confirm pathological conditions or the presence of lymphoproliferative disorder, confirming a new complete response to treatment. With the aim of autologous hematopoietic stem cell transplantation (ASCT), he underwent mobilization of stem cells with the administration of granulocyte growth factor associated with plerixafor (subcutaneously at a dose of 0.24 mg/kg) and subsequent collection of CD34+ cells in two different apheretic sessions (1.23 × 10^6^/kg and 1.29 × 10^6^/kg). Six months after the end of the second line of treatment, maintaining the complete response, ASCT was performed in July 2014, preceded by high-dose chemotherapy according to the FEAM scheme. The total nucleated cells infused were 220.32 × 10^7^, with 80% of mononuclear cells, with a very high value over the 95th percentile in our center’s series, with a median of about 50 × 10^7^ cells/kg. The engraftment of neutrophils (>500/mmc) occurred at day +11 from the day of the infusion, followed by the engraftment of platelets (>20,000/mmc) achieved at day +17. The period of post-chemotherapy aplasia was complicated by the onset of fever accompanied by shaking chills on day +1 from the infusion of stem cells and elevated up to 39.6 °C, poorly responsive to empiric therapy with piperacillin/tazobactam and amikacin. Profuse diarrhea and abdominal discomfort were also present. High-resolution chest computed tomography (CT) scans were performed in search of any infectious foci. Throat, rectal, skin, and nasal swabs for bacterial or fungal pathogens, urine cultures, and blood cultures were performed at peak fever from a peripheral vein and central venous catheter (CVC). A carbapenem-sensitive Klebsiella Pneumonia from the CVC blood culture was found. Based on the tested antibiotic sensitivities, CVC was removed, and antibiotic therapy (metronidazole, vancomycin, and imipenem) was promptly started, with progressive improvement of clinical conditions and the disappearance of febrile and diarrheal episodes. At day +15 from the infusion of stem cells, the white blood cells (WBC) count was 660/mmc, with 840/mmc lymphocytes. Subsequently, without any other noteworthy symptom, five days later, we assisted in a rapid isolated increase of lymphocytes (hemoglobin 9.6 g/dL, platelets 102,000/mmc, and WBC 62,030/mmc, with 2480/mmc neutrophils, 56,450/mmc lymphocytes, and 3050/mmc monocytes). In-depth examinations were performed to search for any infections that might have caused this manifestation. Hepatitis B and C virus, cytomegalovirus, Epstein–Barr virus, Parvovirus B19, HIV, HHV-8, and Toxoplasma gondii test were negative and evaluated via PCR and serological virus investigations. Physical examination of the explorable station did not show any enlarged lymph nodes, also confirmed via a new CT exam. On morphological examination of a peripheral blood smear, lymphocytes with activation characteristics were observed. In contrast, in the blood obtained via marrow aspirate, there were atypical cells with blastic characteristics with a lymphoid habit comprising >50% of the cellularity (Figure 1). A T-lymphocyte population expressing the immunological phenotypes CD3+, CD8+, TCRαβ+, CD2+, CD5+, CD1a−, CD7±, CD38+, and HLA-DR+ was reported on peripheral blood flow cytometry test.

The bone marrow biopsy performed on day +24 was negative for localization of HL and showed a marrow with cellularity less than 20%, with about 50% of small T-lymphocytes (CD3+, CD8 > CD4).

**Figure 2:**
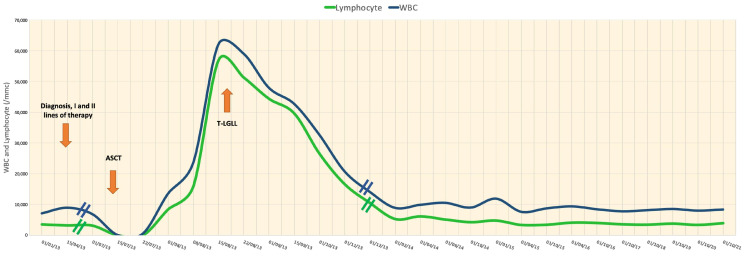
Fluctuation in the values of peripheral lymphocytes, with evidence of the post-ASCT clonal peak progressive spontaneous reduction in the number of these cells.

The T-cell receptor (TCR) clonality analysis from peripheral blood, performed on day +40, was positive for the monoclonal rearrangement in the gamma chain of the TCR gene, consistent with that of a clonal population. To exclude a possible etiological cause of this being T-lymphocytosis or human T-lymphotropic virus type 1 (HTLV-1), viral genome research and a new serological evaluation for common autoimmune disorders were performed (negative in both cases). We chose strict follow-up for idiopathic T-cell clonal lymphoproliferative disorder sustained by T-large granular lymphocytes. At day +50, Hb 11.2 g/dL, platelets 322,000/mmc, WBC 32,790/mmc, and lymphocytes 27,540/mmc were found in the blood count. Flow cytometry confirmed the marked increase in circulating CD3+ T-lymphocytes again. Continuing the follow-up, however, we assisted in a progressive and spontaneous reduction in the number of WBC and lymphocytes (Figure 2), until a definitive normalization occurred at day +250 from the ASCT, with a blood count of Hb 14 g/dL, platelets 399,000/mmc, WBC 8720/mmc, and 4900/mmc lymphocytes, with no presence of a malignant clone found during the new flow cytometry evaluation. Finally, the search for monoclonal rearrangement of the T-cell receptor (TCR) was negative from day +150.

The follow-up continued periodically with visits at first every two months and then annually for 10 years, during that time the patient has maintained a complete response to treatment, confirmed via instrumental examinations (CT and PET), and there is no longer any evidence of the appearance of oligo/monoclonal lymphocytosis, with the last blood count evaluation of Hb 14.5 g/dL, platelets 328,000/mmc, WBC 8380/mmc, and lymphocytes 3940/mmc.

## 3. Discussion

Starting with the literature review, T-LGLL is an infrequent manifestation of HSCT; even rarer is the onset in autologous stem cell transplant (ASCT) patients. Few cases are described in the literature. The first findings are, for example, of patients with non-Hodgkin’s lymphoma (angioimmunoblastic lymphoma) [12] or multiple myeloma [13]. A recent meta-analysis by Awada, H. et al. analyzed the case reports in the literature from 1994 to 2014. A total of 62 cases of suspected post-solid organ transplantation T-LGLL or HSCT were identified. Of them, three were confirmed post-ASCT diagnoses. These patients were affected by diffuse large B-cell lymphoma (DLBCL), chronic lymphocytic leukemia (CLL), and mantle cell lymphoma (MCL). The onset of this condition manifested itself after 7, 2, and 0–2 years from the time of transplant, respectively. All three patients had elevated lymphocytes but no concomitant neutropenia, thrombocytopenia, or anemia and were initiated for follow-up [14].

In the case of negativity in the search for the infection of common viral agents and there being no risk of allogeneic antibody stimulation as observed in donor transplants, the most plausible causal hypothesis lies in chronic antigenic stimulation, which may, in the long-term, lead to clonal expansion, as it may occur in chronic CMV or other virus infections or reactivations. However, T-LGLL is not associated with EBV infection [15]. In all the previously described cases (including Awada’s meta-analysis), the number of clonal T-lymphocytes fluctuated from a minimum of 100/mmc to a maximum of 12,400/mmc, with an average of 4770/mmc [14]. On the other hand, the early recovery of lymphocytes is a prognostic factor for superior post-ASCT survival for both diffuse large B-cell lymphoma and multiple myeloma patients. In this regard, it is ascertained that the number of infused lymphocytes significantly influenced lymphocyte recovery following ASCT [16,17,18].

A possible pathogenetic mechanism could be found in the complex immune dysregulation after stem cell infusion, with a cytokine storm able to lead to endothelium damage [19] and constitutive activation of the JAK-Stat3 signaling pathway responsible for the transcriptome of large T-lymphocytes [10]. Finally, the uncontrolled proliferation of these cells could be due to a reaction caused by alloantigen stimulation concomitant with graft-versus-host disease (GVHD), in response to infectious agents in a context of immunodeficiency, or relapse of a pre-existing autoimmune process [11].

The case described here is of particular interest. No descriptions of T-LGLL in the literature arise for a patient undergoing ASCT for Hodgkin’s lymphoma who had been previously splenectomized. The impressive number of clonal T-lymphocytes (greater than 60,000/mmc) is also of considerable interest, far more significant than in the previous cases reported in the literature. In this regard, it should be considered that splenectomy may determine an abnormal lymphocyte distribution and lead to a different antigenic exposure of the immune system, considering the negative histological exam reported after the splenectomy. In our opinion, the very high number of lymphocytes infused may also have played a role in establishing the elevated lymphocyte count in PB.

Concerning our treatment choice, we decided to use active surveillance and a “watch and wait” strategy, which led to the spontaneous resolution and disappearance of the clonal cellular component during follow-up and was maintained for an extended period. This aspect has never been observed before, to our knowledge. In any case, despite the absence of clear therapeutic guidelines, valid alternatives could be methotrexate (10 mg/m^2^ per week), cyclophosphamide (100 mg per day), or ciclosporin A (3 mg/kg per day) as these immunosuppressive drugs widely used, with an overall response ratio superior to 45% of patients in 10 retrospective studies. In case of relapse/refractory, purine analogs (fludarabine, cladribine, or bendamustine) are the standard choice. Polychemotherapy schemes (CHOP, cytosine arabinoside-based scheme) are toxic and should be reserved for use only in the case of aggressive leukemia [20]. Emerging therapies are based on targeted molecules (e.g., JAK/Stat pathway, IL-2, and IL-15 inhibition), and are currently under clinical trial evaluation [21].

Finally, after a response to treatment is achieved, full blood count, abdomen, and lymph nodes ultrasound exams, and physical exam, should be performed to monitor the follow-up adequately, with the possibility of supportive care considered for patients suffering from anemia or neutropenia using erythropoietin or granulocyte colony-stimulating factor [10].

## 4. Conclusions

T-LGLL is a rare post-transplant complication. The mechanisms involved are still unclear and require further study, needing accurate answers to describe them. In the context of HSCT, a proper pre-transplant workup could help to find small cell clones able to proliferate under conditions of immunosuppression. Despite this, it is possible that this post-transplant lymphocytosis could be transient, guaranteeing an excellent result with a watch-and-wait approach.

## Data Availability

Data are available from the authors upon request.
